# Accelerating bioinformatics research with International Conference on Intelligent Biology and Medicine 2020

**DOI:** 10.1186/s12859-020-03890-y

**Published:** 2020-12-28

**Authors:** Yan Guo, Li Shen, Xinghua Shi, Kai Wang, Yulin Dai, Zhongming Zhao

**Affiliations:** 1 Department of Internal Medicine, Comprehensive Cancer Center, University of New Mexico, Albuquerque, NM 87131 USA; 2 Department of Biostatistics, Epidemiology and Informatics, Perelman School of Medicine, University of Pennsylvania, Philadelphia, PA 19104 USA; 3 Department of Computer and Information Sciences, College of Science and Technology, Temple University, Philadelphia, PA 19122 USA; 4 Raymond G. Perelman Center for Cellular and Molecular Therapeutics, Children’s Hospital of Philadelphia, Philadelphia, PA 19104 USA; 5 Center for Precision Health, School of Biomedical Informatics, The University of Texas Health Science Center at Houston, Houston, TX 77030 USA

**Keywords:** Network, Imaging, Machine learning, Gene expression

## Abstract

The International Association for Intelligent Biology and Medicine (IAIBM) is a nonprofit organization that promotes intelligent biology and medical science. It hosts an annual International Conference on Intelligent Biology and Medicine (ICIBM), which was initially established in 2012. Due to the coronavirus (COVID-19) pandemic, the ICIBM 2020 was held for the first time as a virtual online conference on August 9 to 10. The virtual conference had ~ 300 registered participants and featured 41 online real-time presentations. ICIBM 2020 received a total of 75 manuscript submissions, and 12 were selected to be published in this special issue of BMC Bioinformatics. These 12 manuscripts cover a wide range of bioinformatics topics including network analysis, imaging analysis, machine learning, gene expression analysis, and sequence analysis.

## Introduction

The International Conference on Intelligent Biology and Medicine (ICIBM 2020) was organized and hosted by the International Association for Intelligent Biology and Medicine (IAIBM), University of Philadelphia, and Temple University. It was held on August 9–10 2020. The originally scheduled location was moved from Philadelphia to online due to the COVID-19 pandemic. The conference had 300 registrants and 291 attended online. It received 75 original manuscripts for consideration of oral presentations and special issues. 41 authors presented their researches in live sessions over ZOOM online conference platform. These 75 manuscripts went through rigorous review processes, and 12 high-quality manuscripts were selected to represent the bioinformatics aspect of the conference. All 12 selected manuscripts went through further review and revision process before finally publishing in the ICIBM 2020 BMC Bioinformatics special edition. This special issue has a strong focus on network analysis, with other topics including imaging, machine learning, gene expression, and sequence analysis.

## Network analysis

Network analysis is a set of integrated techniques to depict relationships among entities and to analyze the structure and patterns that emerge from these relations. Network analysis has a long history of application in biomedical researches. This special issue contains five manuscripts that leveraging network analysis techniques in biomedical research. Wen et al. conducted research titled “Clinical connectivity map for drug repurposing: using laboratory tests to bridge drugs and diseases” [1]. In this study, the authors proposed a clinical connectivity map framework for drug repurposing by leveraging laboratory tests to analyze complementarity between drugs and diseases. By evaluating 392 drugs for 6 chronic diseases, multiple hidden drug-disease associations were identified. Ha et al. published “Compositional zero-inflated network estimation for microbiome data” [2]. In this study, the authors proposed the COmpositional Zero-Inflated Network Estimation (COZINE) method for inference of microbial networks which addresses these critical aspects of the data while maintaining computational scalability. COZINE relies on the multivariate Hurdle model to infer a sparse set of conditional dependencies which reflect not only relationships among the continuous values, but also among binary indicators of presence or absence and between the binary and continuous representations of the data. Through simulation, the authors showed that COZINE had better performance in capturing the various types of microbial relations than existing methods. Li et al. published “Effect of APOE ε4 on multimodal brain connectomic traits: a persistent homology study” [3]. In this work, the authors proposed a novel multimodal brain network modeling framework and a network quantification method based on persistent homology for identifying APOE ε4-related network differences. The authors found that their method outperformed existing methods and yielded connectomic patterns specific APOE ε4 carriers and non-carriers. Zhou et al. carried out a research study titled “LDscaff: LD-based scaffolding of de novo genome assemblies” [4]. In this study, the authors proposed the method LDscaff for drafting genome assembly incorporating linkage disequilibrium information. Evaluation of LDscaff from both simulated and real data showed substantial improvement. For example, the donkey genome assembled by LDscaff had an improved N50 length of 32.1 Mb from 23.8 Mb. Liu et al. reported gene co-expression network method in their paper entitled “TPSC: a module detection method based on topology potential and spectral clustering in weighted networks and its application in gene co-expression module discovery” [5]. In this study, the authors proposed a novel module detection algorithm TPSC based on topology potential and spectral clustering algorithm to detect co-expressed modules. Through testing the method on real data, the authors found that TPSC was capable to detect more size-balanced and granular modules. Moreover, TPSC can be applied to any generally fully connected and weighted networks. Mandal et al. published “In silico ranking of phenolics for therapeutic effectiveness on cancer stem cells” [6], a study to rank cancer stem cell genes for alternative cancer treatment. Weighted bipartite graphs were constructed from 1118 cancer stem cell genes along with their interacting phytochemicals from phenolic group. A ranking technique was developed based on PageRank (PR) algorithm for ranking the phenolic group. The results suggested that some phenolics are potential molecules for cancer stem cell-related cancer treatment.

## Imaging and machine learning

Imaging techniques have been widely used in biomedical researches. Machine learning is the study of computer algorithms that improve automatically through experience. Machine learning is one of the most used techniques in computational biology. In this special issue, four studies covering imaging and machine learning analysis were included, two of them applied neural network-based deep learning techniques in imaging analysis. Tu et al. published “Fingerprint restoration using Cubic Bezier Curve” [7], which is a study about restoring partial fingerprint. In this study, the authors modeled fingerprints with Bezier curves and proposed a novel algorithm to detect and restore fragmented ridges in fingerprints. The evaluation showed that the false-positive rate was 4.59% and the false-negative rate was 2.83% which was a substantial improvement from the previous methods. Al-Azzawi et al. wrote the manuscript “Auto3DCryoMap: an automated particle alignment approach for 3D cryo-EM density map reconstruction” [8]. The authors investigated 3D density map reconstruction from cryogenic electron microscopy images and proposed a fully automated cryo-EM 3D density map reconstruction approach Auto3DCryoMap based on deep learning particle picking. It uses deep learning approach to automatically pick the particles from the micrographs and classify them into top view or side-view. Instead of increasing the signal-to-noise ratio by using 2D class averaging, Auto3DCryoMap uses the perfect 2D mask to produce locally aligned particle images. Extensive evaluations showed that the Auto3DCryoMap can accurately align structural particle shapes and can construct a decent 3D density map from only a few thousand aligned particle images while the existing tools require hundreds of thousands of particle images and reconstruct a better 3D density map. Jo et al. published “Deep learning detection of informative features in tau PET for Alzheimer’s disease classification” [9], in which the authors developed a deep learning-based framework to identify informative features for Alzheimer’s disease classification using tau position emission tomography scans. By applying five-fold cross-validation, the authors demonstrated their method yielded an accuracy of 90.8%. Zeng et al. published “Deep learning for cancer type classification and driver gene identification” [10], a study about cancer driver gene identification. The authors developed DeepCues, a deep learning model that utilizes convolutional neural networks to unbiasedly derive features from raw cancer DNA sequencing data. Raw whole-exome sequencing features, germline variants, and somatic mutations, including insertions and deletions, were interactively amalgamated for feature generation. DeepCues was applied to a dataset from The Cancer Genome Atlas to classify seven different types of major cancers. The authors obtained an overall accuracy of 77.6%. By comparing DeepCues with conventional methods, the authors demonstrated a significant overall improvement.

## Gene expression

De Torrente et al. conducted a study titled “The shape of gene expression distributions matter: how incorporating distribution shape improves the interpretation of cancer transcriptomic data” [11]. In this study, the authors examined the gene expression from the unique perspective of statistical distribution and found that less than 50% of all genes were normally distributed. These non-normally distributed genes had strong prognostic values. This study highlights the value of studying gene distribution shape to model heterogeneity of transcriptomic data.

## Sequence analysis

Sequence analysis is the most common bioinformatics work due to the popularity of high throughput sequencing technology. In this supplement issue, Liu et al. conducted a study about short tandem repeat (STR) titled “Genome-wide detection of short tandem repeat expansions by long-read sequencing” [12]. STR, one type of “microsatellite” markers, is a tract of repetitive DNA in which certain DNA motifs (typically < 10 base pair long) are repeated multiple times in a genomic region. The normal ranges of repeat counts for most STRs in human populations are not well known, preventing the prioritization of STRs that may be associated with human disease. In this study, the authors used RepeatHMM to infer normal ranges of 432,604 STRs using 21 human genomes by whole-genome long-read sequencing technologies. The results were curated into a database, RepeatHMM-DB which is expected to facilitate large-scale prioritization and identification of disease-relevant tandem repeats for patients with undiagnosed diseases that may be caused by repeat expansions.

## Conclusions

ICIBM is an annual international conference, which has been held every year since 2012 (except 2017). It promotes a highly interactive and friendly platform for both young and senior researchers to exchange their research, foster collaboration, as well as expand educational activities. Due to the COVID19 pandemic, ICIBM 2020 was held online for the first time with 291 attendees from around the world. Of the 75 submitted manuscripts, we selected 12 manuscripts that describe innovative, computational work for this BMC Bioinformatics special issue. We expect these manuscripts to promote further investigation in the same or similar topics, and lead to more research toward translational clinical applications.
